# Molecular Identification and Targeted Quantitative Analysis of Medicinal Materials from *Uncaria* Species by DNA Barcoding and LC-MS/MS

**DOI:** 10.3390/molecules24010175

**Published:** 2019-01-04

**Authors:** Shugen Wei, Zuliang Luo, Shengrong Cui, Jing Qiao, Zhonglian Zhang, Lixia Zhang, Jine Fu, Xiaojun Ma

**Affiliations:** 1 Guangxi Botanical Garden of Medicinal Plants, Nanning 530023, China; weishugen2@163.com; 2 Institute of Medicinal Plant Development, Chinese Academy of Medical Sciences & Peking Union Medical College, Beijing 100193, China; zuliangluo@163.com (Z.L.); c1061729635@163.com (S.C.); qiaojing_happy@126.com (J.Q.); 3 Yunnan Branch, Institute of Medicinal Plant Development, Chinese Academy of Medical Sciences, Jing Hong 666100, China; zzl0605@163.com (Z.Z.); 13988194288@163.com (L.Z.)

**Keywords:** *Uncaria* species, identification, quantitative analysis, DNA barcoding, LC-MS/MS.

## Abstract

The genus *Uncaria* is an important source of traditional Chinese medicines with multiple therapeutic effects. The identification of the correct species and accurate determination of the contents of bioactive constituents are important for quality control of *Uncaria* medicinal materials. Here, an integrated evaluation system based on DNA barcoding for species identification and quantitative analysis by LC-MS/MS has been established. DNA barcoding based on the ITS2 barcode region showed sufficient discriminatory power to precisely identify 24 samples from seven *Uncaria* species. The length of the 24 ITS2 sequences of *Uncaria* samples is 227 bp, and 17 variation sites were detected. Additionally, the results of qualitative and quantitative chemical analyses by LC-MS/MS indicated that the chemical compositions of all *Uncaria* samples were similar; while their contents of targeted alkaloids in samples from different species and origin areas were different. The contents of rhynchophylline (RC) and isorhynchophylline (IRC) were 2.9–1612 mg/kg and 2.60–1299 mg/kg in all tested samples, respectively. This study concludes that DNA barcoding should be used as the first screening step for *Uncaria* medicinal materials. Then, integration of DNA barcoding with chemical analyses should be applied in quality control of *Uncaria* medicinal materials in the medicinal industry.

## 1. Introduction

The pantropical plant genus *Uncaria* contains about 34 species. The majority are widely used as traditional medicines worldwide for the treatment of fevers, headaches, gastrointestinal illness, hypertension, epilepsy, wounds and ulcers, etc. [1,2,3]. Uncariae Ramulus Cum Uncis (URCU), the dried hook-bearing branches of five plants—*Uncaria rhynchophylla* (URH), *Uncaria hirsute* (UHI), *Uncaria macrophylla* (UMA), *Uncaria sinensis* (USI) and *Uncaria sessilifructus* (USE)—is a well-known herbal medicine mentioned in Chinese Pharmacopoeias [4]. Many other *Uncaria* species have also been circulated in local herbal markets in China as substitutes. In the Japanese pharmacopoeias, URCU was defined as hook or the hook-bearing stem of three plants: URH, UMA, and USI [5]. The phytopharmaceuticals that contain *Uncaria* plants, such as Tian-ma-gou-teng Granules, Diao-teng-san (Cho-Deung-Sanin Korean and Choto-san in Japanese) and Yi-Gan san (Yokukansan in Japanese) have become best-selling herbal medicines for the treatment of strokes, hypertension, chronic headaches and dizziness in China, Korea and Japan [6]. Yokukansan has been approved by the Ministry of Health, Labor, and Welfare of Japan as a remedy for neurosis, insomnia, and irritability in children [3].

As the medicinal uses of *Uncaria* species have increased, a great deal of studies concerning the phytochemistry and pharmacology have been carried out. More than 200 chemical constituents, including indole alkaloids, triterpenes, flavonoids and phenylpropanoids, etc. have been isolated from the genus *Uncaria*. Of these, indole alkaloids are regarded as the main bioactive constituents responsible for the broad spectrum of biological properties reported for *Uncaria* species. There have been about 120 indole alkaloids phytochemically isolated from the genus *Uncaria* [7]. Although so many constituents are found, most research has focused on a relatively small number of compounds such as rhynchophylline (RC), isorhynchophylline (IRC), corynoxeine (CX), isocorynoxeine (ICX), hirsutine (HTI), hirsuteine (HTE), corynoxine (CN), corynoxine B (CNB), etc, particularly the constituents RC and IRC that show high contents in *Uncaria* plants.

Pharmacological studies have demonstrated that the bioactivities of the different alkaloids in these plants display significant variability. RC and IRC are the main antihypertensive components of that expand peripheral blood vessels, and the effect of IRC was stronger than that of RC [8]. RC acts on cardiovascular and central nervous system diseases, and its mechanisms have been related to ion channel regulations as well as neurotransmitter transport and metabolism [9]. HTI has cardioprotective effects against cardiomyocyte injury induced by hypoxia and negative chronotropic and antiarrhythmic activity [10]. Studies have shown that RC and IRC are unstable and decrease with the increase of storage time. Moreover, RC and IRC are affected by heating, and they can be transformed into each other or decomposed into other alkaloid derivatives [8,11,12]. In addition, there are large variations in the contents of these bioactive alkaloids in *Uncaria* samples according to the species and different origin areas [6,13]. Therefore, the source, processing and storage will affect the quality and therapeutic efficacies of URCU. It is noteworthy that the current edition of the Chinese Pharmacopoeia does not stipulate the contents of active ingredients in URCU [4]. 

DNA barcoding is a species identification system that uses short DNA sequences of a standardized gene region [14,15]. It can accurately identify the species with similar morphological characteristics and chemical compositions. DNA barcodes are widely used in the identification of medicinal plants due to their simple operation and high repeatability. Many *Uncaria* plants are morphologically similar, making identification of their origin species difficult. Our previous studies showed that ITS2 is most suitable as a DNA barcode candidate for identification of medicinal plants of the genus *Uncaria* [16]. Therefore, in our study ITS2 was used to establish a suitable DNA barcoding protocol to identify *Uncaria* species. In addition, many approaches have recently been developed for the qualitative and quantitative analysis of the major alkaloids in URCU. Among these, HPLC and LC-MS/MS have been most frequently reviewed elsewhere [1,6,17,18]. Although they have contributed significantly to the current state of knowledge of indole alkaloids in URCU, they have their own drawbacks for the quality evaluation of URCU, such as a focus on quantifying just a few compounds or needing excessively long elution times. 

Here, we propose the use of DNA barcoding technology based on previous studies to identify the raw material used as URCU. After the initial identification step, we are trying to develop a simple, fast method for simultaneously determine eight bioactive alkaloids in *Uncaria* samples. Our results suggest that DNA barcoding could be used as a screening step during the herbal medicine manufacturing process, and further quantitative analysis is important for improving the quality of URCU. This study should be helpful in the development of strategies for the conservation, utilization and quality control of URCU.

## 2. Results

### 2.1. DNA Barcoding Analysis

ITS2 sequences of all 24 *Uncaria* samples were all successfully amplified by the universal primers ITS2F/3R. Sequence analysis showed that 24 ITS2 sequences had the same 227 bp length and the average GC content was 65.6%. There were 17 variable sites in the ITS2 sequences that were segregated into nine sequence haplotypes (H): H1 (UHI1), H2 (UHO1), H3 (UMA1, UMA3), H4 (UMA2), H5 (URH1-4 and URH6-15), H6 (URH5), H7 (USC1), H8 (USE1) and H9 (USI1 and USI 2) (Table 1). Moreover, the Kimura 2-parameter (K2P) distances among them had a mean value of 0.017, ranging from 0.003 to 0.037.

BLAST and NJ tree methods were used to authenticate the 24 *Uncaria* samples. All ITS2 sequences were aligned using the BLAST algorithm to search for the highest sequence similarity amongst samples to identify the species based on the online databases (http://blast.ncbi.nlm.nih.gov/Blast.cgi). The results indicated H1, H3, H4, H5, H7, H8 and H9 shared 100% homology with *Uncaria hirsute* (MF033334.1), *Uncaria macrophylla* (MF033344.1), *Uncaria macrophylla* (KF881258.1), *Uncaria rhynchophylla* (MG730347.1), *Uncaria scandens* (KF881263.1), *Uncaria sessilifructus* (MF033339.1) and *Uncaria lancifolia* (KF881263.1), respectively. H2 shared 99% identity with *Uncaria homomalla* (MF033292.1) and contained three SNP sites at position 23, 58 and 178, thus were identified as *Uncaria homomalla* (Figure S1). Unexpectedly, H5 sample showed overlapped ITS SNP signals at sites 25, 62, 149, 196 and 219. Since the ITS region was located on nrDNA and inherited from both parents, this sample should be considered a hybrid species. 

**Figure 1:**
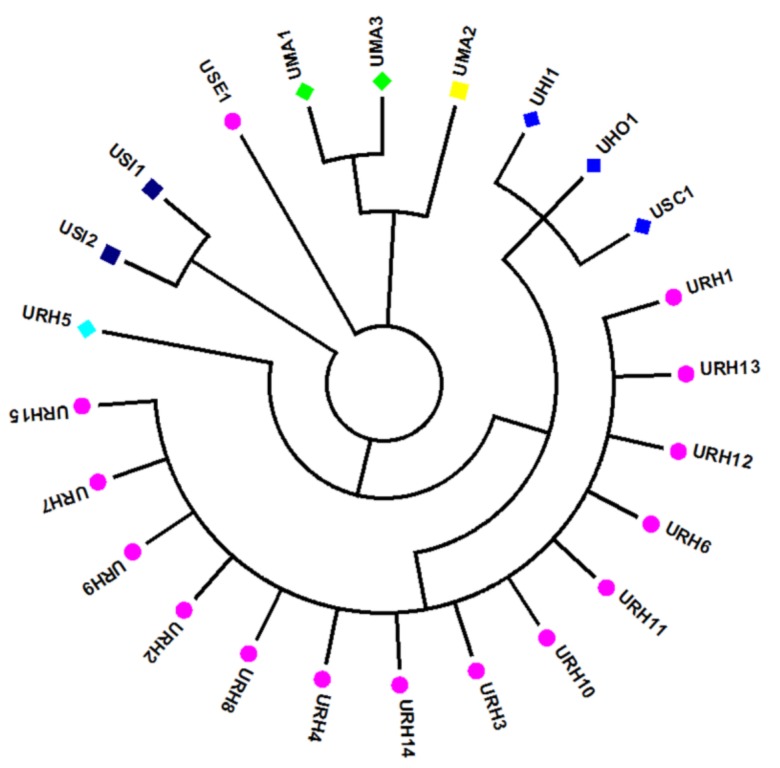
Results of NJ-tree analysis based on ITS sequences of the 24 *Uncaria* samples.

To identify the species of the 24 *Uncaria* samples more accurately and visually, a phylogenetic tree was constructed based on the ITS sequences. NJ tree analysis was consistent with the BLAST result. Samples from the same species origins had similar ITS2 sequences and were in the same clusters. All samples were divided into six main characteristic clusters (Figure 1). Among them, UHI, UHO and USC were divided into one branch and the similarity among them was 99%. The genotype of ITS2 sequences in UHO and USC at 23bp, 45bp and 59bp were ATC and CTT, respectively, but, the genotype of ITS sequences in UHI at the same locus were all C, based on this, it also able to identify them accurately. DNA barcoding could successfully identify the *Uncaria* samples.

### 2.2. LC-MS/MS Analysis

#### 2.2.1. Method Validation

In order to confirm that the developed analytical method employed is reliable, adequate and consistent, method validation was carried out, and the results are listed in Table S2. Good linearity with a square root of the correlation coefficient (r) from 0.9990 to 0.9997 was achieved within the investigated ranges for all targeted alkaloids. The LODs of the eight targeted alkaloids is within the range of 0.02–0.08 ng/mL, and LOQs is within the range of 0.05–0.2 ng/mL, indicating high sensitivity of the method with these chromatographic and MS/MS conditions. The relative standard deviations (RSDs) of the intra-day and inter-day for all the investigated components, were less than 5.9% and 5.6% respectively. High recovery values fell within the range 81.6–104% at three different levels of concentration and the RSDs were less than 5.2%. Which demonstrated that the method was precise and accurate for simultaneous quantitative evaluation of eight targeted alkaloids in the *Uncaria* samples. The repeatability and stability presented for all targeted alkaloids were not more than 5.6%.

The matrix effects (ME) for each targeted alkaloid were calculated and the results (Table S2) showed that slight signal enhancement or signal suppression was observed, the ME for the different analytes were between 87.3% and 104.5%. Therefore, we suggest that the *Uncaria* matrices would not interfere with the accurate determination of all targeted alkaloids using the developed LC–MS/MS method. These results indicated that the developed method was acceptable for quantitative analysis of the targeted alkaloids in the *Uncaria* samples.

#### 2.2.2. Quantitative Analysis of Real Samples

**Figure 2:**
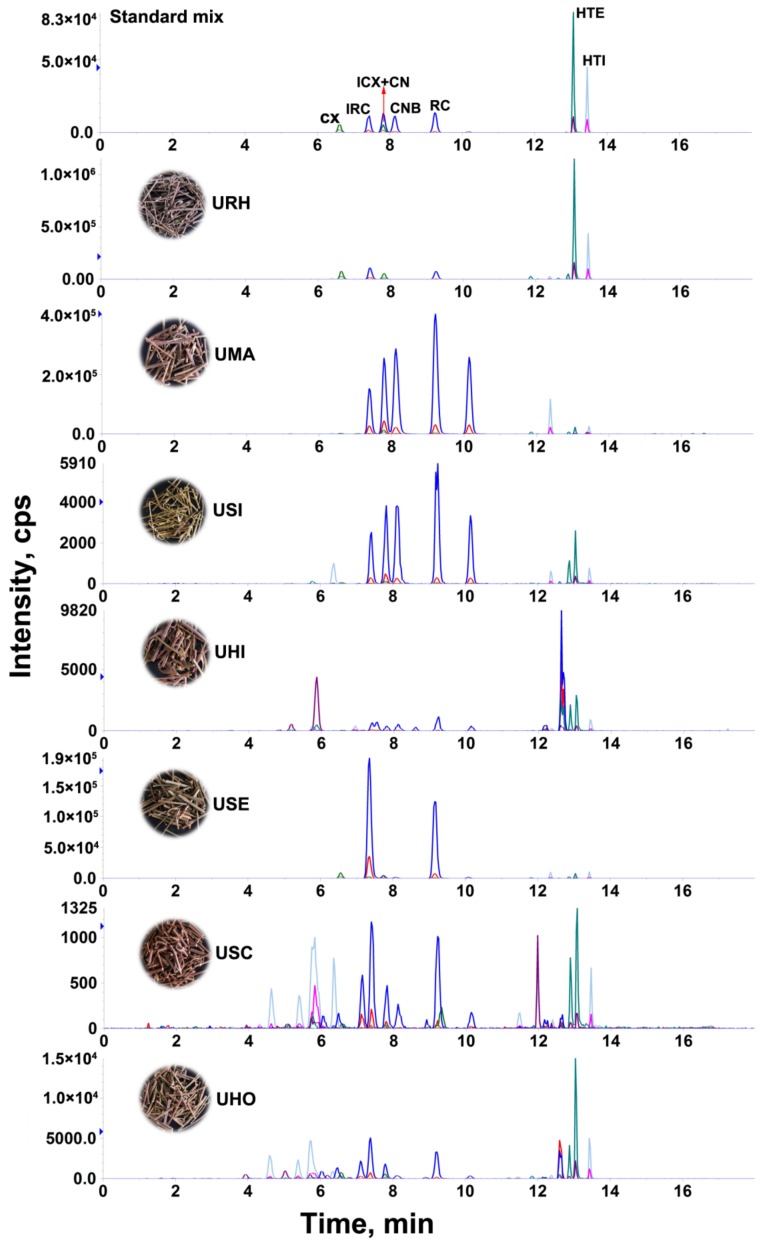
The MRM chromatograms of standard mixture and representative *Uncaria* samples.

The developed LC-MS/MS method was applied to determine the content of eight targeted alkaloids in 24 batches of *Uncaria* samples collected from different regions of Guangxi Province, China. The targeted alkaloids were identified by comparison of their retention times, precursor and product ions. The quantitative analyses were performed by mean of external standard method and the results are summarized in Table 2 and the MRM chromatograms of representative samples is shown in Figure 2, where it can be observed that the contents of each targeted alkaloid and the total contents varied significantly in the different samples. The total contents of targeted alkaloids in the range of 12.4–5330.6 mg/kg from 24 batches of *Uncaria* samples. In the species URH, UMA and USE, the total contents of alkaloids is higher than 1 g/kg, while in the species USI, UHO, USC and UHI, the total contents of alkaloids is lower than that of 0.1 g/kg.

In terms of individual constituents, there were large variations in the contents of these targeted alkaloids in the *Uncaria* samples according to the species and different origin areas. RC (10.6–232.6 mg/kg), IRC (27.7–455.4 mg/kg), CX (27.5–728.0 mg/kg), ICX (18.8–551.4 mg/kg), HTI (146.9–945.7 mg/kg) and HTE (145.2–1156.3 mg/kg), are abundant, but CNB is not detected and CN is low, even at trace levels in the URH samples. As the species with the highest alkaloids content, RC, IRC, CN and CNB are abundant in UMA (1144.0–1612.0 mg/kg for RC, 623.9–1299.4 mg/kg for IRC, 1057.6–1088.4 mg/kg for CN, and 1033.5–1332.6 mg/kg for CNB). For the species of USI, UHO, USC and UHI, the contents of each targeted alkaloid is low even trace. According to the 100% stacked bar graphs (Figure S2), we can see that HTI and HTE are the main components and their contents account for 32–94% in total content of all targeted alkaloids in the URH samples. Among the remaining six species, the RC and IRC are the main components and they contents account for 41–88% in total contents of targeted alkaloids, especially in USE, the proportion reached 88%.

**Figure 3:**
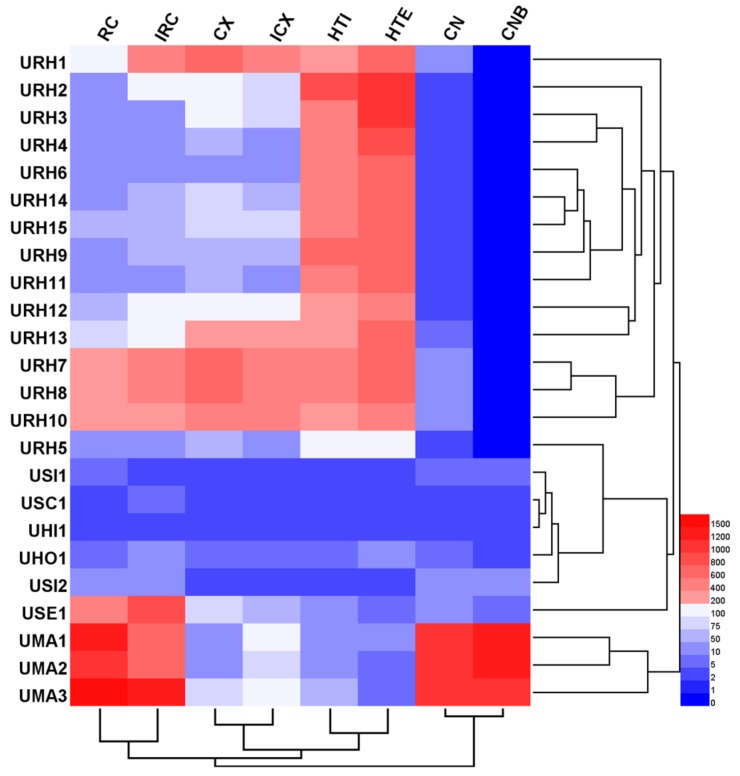
Hierarchical clustering analysis of *Uncaria* samples.

Hierarchical clustering analysis (HCA), a chemical pattern recognition and classification evaluation method, was used to assess the resemblance and differences of 24 Uncaria samples. HCA was carried out based on the contents of targeted alkaloids using a software of HemI (Heatmap Illustrator, version 1.0. The CUCKOO Workgroup, Wuhan, China. http://hemi.biocuckoo.org/index.php). Between-groups linkage clustering was chosen and the Euclidean distance was selected to assess the resemblance of 24 samples, and then classify them into groups. The resulting hierarchical cluster heat-map is shown in Figure 3, from which the quality characteristics were revealed more clearly. According to the results, all samples were classified into three main clusters. Except for URH5, 14 samples belong to URH species was formed cluster I. Additionally, cluster I was divided into three groups based on the contents of targeted alkaloids. In cluster I, all URH samples gathered from different or same origin areas showed that they quality characteristics were different. This might be due to the differences in harvesting time, storage time, storage conditions, and other factors. Cluster II including seven samples belonged to URH, USI, USC, UHI, UHO and USE. The samples of URH5 and USE1 were divided into different groups, respectively. The remaining samples including UMA1, UMA2 and UMA3 belonged to UMA were put into cluster III. The distances between samples in the same group were shorter than distances between samples not in the same group, indicating that internal qualities were more similar within groups compared to samples in other groups. These results agreed with the DNA barcoding analysis findings.

**Figure 4:**
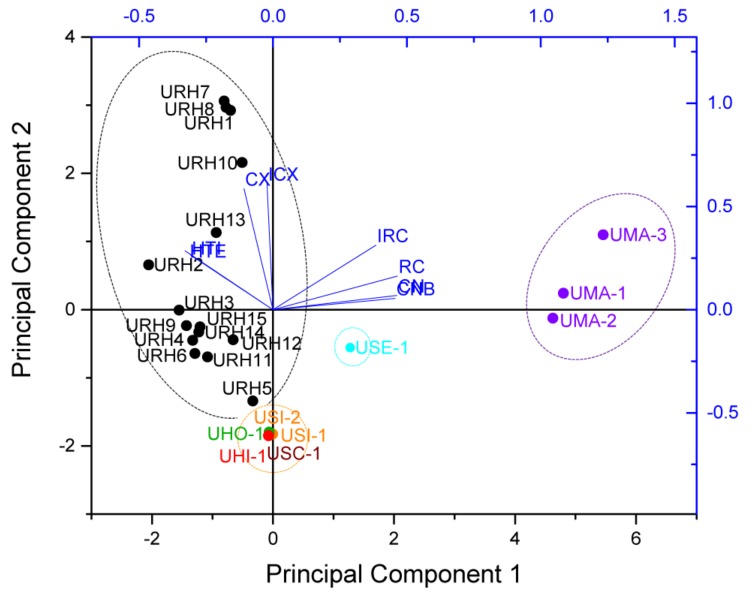
The score plot and loading plot of PCA for different *Uncaria* samples.

Principal component analysis (PCA) is a multivariate method and widely used in data analysis to summarize variation, which is implemented as a data-reduction technique to generate a visual scatter plot for the qualitative evaluation of resemblances and differences between the studied samples [19]. In order to amplify the difference in the samples and easily discriminate the samples, the PCA was carried out based on the contents of targeted alkaloids, which consisted of a matrix of 8 × 24, with each row containing the content values of targeted alkaloids and each column representing a sample. Then, the variables were centered and scaled to “Unit Variance” before performing the PCA, the results show that the first two principal components (PC1/PC2) explained 81.7% of the total variance. According to the scores plot of PCA, all samples were divided into four groups according to the content values of targeted alkaloids (as shown in Figure 4). Group 1 contained 15 samples (URH1–URH15) belonging to URH species. Group 2 contained three samples (UMA1–UMA3) belonging to UMA. Group 3 contained five samples belonging to USI, UHO, USC and UHI. Group 4 contained one sample belonging to USE. It suggested that they were associated with similar internal quality in same group. The loading plot of PCA based on the targeted alkaloids showed that the HTI and THE contributed most to URH, followed by CX and ICX. While RC, IRC, CN and CNB contributed most to UMA. The classification results of PCA adequately showed the noticeable species, geographical and chemical differences among the samples. The results obtained from PCA based on targeted alkaloids of *Uncaria* samples were in accordance with that of HCA.

## 3. Discussion

Overall, the present study showed that the samples belonging to seven *Uncaria* species varied at a genetic level, and these species could be identified based on the ITS2 sequences. Their chemical compositions were similar, but the contents of targeted alkaloids in samples collected from different species and origin areas were different. Exclude the above species and habitats factors, this result might due to the differences of harvesting time, storage time and condition, and other factors. According to previous studies, the contents of RC and IRC were changed with growth time [18]. The results showed that RC decreased from 5.1% to 0.9% and IRC decreased from 8.2% to 6.0% in *Uncaria* extracts after six months storage at room temperature [11]. The thermal stability of total alkaloids in *Uncaria rhynchophylla* was investigated in a previous study, and the results indicated that IRC was unstable to heating, and when heated in 60 °C and 90 °C for 2 h, IRC content decreased more than 38% and 50%, respectively [12]. Moreover, the phytopharmaceutical studies demonstrated that bioactivities of the different alkaloids in *Uncaria* plants display significant deviations. Therefore, we propose the use of DNA barcodes as a powerful first screening step. Then, an accurate LC-MS/MS method for quality control of *Uncaria* samples is necessary to ensure the quality of medicinal materials and effect of clinical medication. In future research, even this evaluation system based on DNA barcoding for identification and LC-MS/MS for quantification should be widely used in various links of medicinal industry. For example, high quality species and varieties selection based on DNA identification for standardized planting, the confirming of optimum harvest time, storage time and condition based on bioactive components analysis for *Uncaria* medicinal materials, quality control of raw materials based on DNA barcoding and LC-MS/MS for traditional Chinese medicine decoctions and pharmaceutical manufacturers, etc.

## 4. Materials and Methods 

### 4.1. Chemicals, Reagents, and Herbal Materials

Standards of eight targeted alkaloids, including rhynchophylline (RC), isorhynchophylline (IRC), corynoxeine (CX), isocorynoxeine (ICX), hirsutine (HTI), hirsuteine (HTE), corynoxine (CN) and corynoxine B (CNB) (Table S1) were obtained from Chengdu Must Bio-Technology Co., Ltd. (Chengdu, China) and Shanghai Yuanye Bio-Technology (Shanghai, China). The purities of these reference compounds were all over 98.0%. HPLC-grade acetonitrile, methanol, formic acid were purchased from Thermo Fisher Scientific (Fair Lawn, NJ, USA). Other reagents and chemicals were of analytical grade and bought from Sinopharm Chemical Regent Beijing Co., Ltd (Beijing, China). Water was obtained using a Milli-Q water purification system (Millipore, Milford, MA, USA).

The 24 samples of seven Uncaria species—*Uncaria rhynchophylla* (URH), *Uncaria hirsute* (UHI), *Uncaria macrophylla* (UMA), *Uncaria sinensis* (USI), *Uncaria sessilifructus* (USE), *Uncaria homomalla* (UHO) and *Uncaria scandens* (USC)—were collected from Guangxi Province in China. They were identified as genuine samples of *Uncaria* species by Prof. Xiaojun Ma (Institute of Medicinal Plant Development, Chinese Academy of Medical Sciences and Peking Union Medical College, Beijing, China). All samples were dried at room temperature (22–25 °C) and stored in an acclimatized and humidity free room. All samples were pulverized and homogenized, sealed in ziplock bags, and stored at room temperature before analysis. The voucher specimens were kept in the medicinal herbs reference library at the Institute of Medicinal Plant Development.

### 4.2. DNA Barcoding Analysis

#### 4.2.1. DNA Extraction, PCR and Sequencing

Total genomic DNA was extracted from 24 Uncaria samples (∼100 mg) according to the instructions of the plant DNA extraction kit (Tiangen Biotech Co., Beijing, China). All the DNA samples were stored at −20 °C before analysis. The ITS2 regions of nuclear DNA were amplified using the universal primers (ITS2F/3R) according to previously described methods [20]. The PCR mixture (30 µL) contained template DNA 2 µL, forward primer (10 µM) 1.2 µL, reverse primer (10 µM) 1.2 µL, Mix-Taq enzyme 15 µL and ddH2O 10.6 µL. The PCR conditions were 94 °C for 2 min, followed by 30 cycles at 94 °C for 30 s, 55 °C for 30 s and 72 °C for 1 min, with a final incubation at 72 °C for 10 min. PCR-amplified products were examined by 1.0% agarose gel electrophoresis before sequenced by the Sino GenoMax Co., Ltd (Beijing, China). To ensure the accuracy, samples were all sequenced in two-way mode.

#### 4.2.2. Sequence Alignment and Analysis

CodonCode Aligner V3.7.1 (CodonCode Co., Dedham, MA, USA) was performed to assemble raw data, delete low-quality regions, and remove 5.8 S and 28 S sections at both ends of the sequences. Sequences were aligned using DNAman (version 8.0, Lynnon Biosoft, San Ramon, CA, USA), and SNPs were identified by visual inspection of the alignments. MEGA6.0 was employed for the final ITS2 sequence characteristic analysis. Sequence lengths, G + C content, and genetic distances were calculated. Then were submitted to GenBank-NCBI for comparison with the deposited sequences using the tool BLAST. All of the samples and sequences from database were selected to construct a neighbor-joining tree (NJ tree). Bootstrap tests were conducted using 1000 replicates to estimate the identification efficacy of phylogenetic relationships.

### 4.3. LC-MS/MS Analysis

#### 4.3.1. Samples and Standard Solution Preparation

A dried powdered sample (0.2 g, passed through a 500 µm mesh sieve) was precisely weighed and extracted with methanol/water (20 mL, 70:30, *v/v*) by ultrasonic extraction at room temperature for 30 min. After centrifugation (4000 rpm/10 min), 1 mL of supernatant was diluted to 10 mL with methanol/water (70:30, *v/v*) in a volumetric flask, filtered (0.22 μm membrane filter) and stored at 4 °C out of light before analysis. The standards of the eight targeted alkaloids were accurately weighed, transferred to volumetric flasks, and dissolved in methanol to make individual stock solutions. Then, each stock solution was mixed to prepare a final mixed stock solution containing eight standards, which was stored in a refrigerator at 4 °C. For analysis, the mixed solution was diluted by methanol/water (70:30, *v/v*) to obtain a series of working solutions of different concentrations.

#### 4.3.2. Instrumentation and Analytical Conditions

The HPLC system consisted of two LC-20AD pumps, a DGU-20 A3 degasser, an SIL-20AC autosampler and a CTO-20A column oven (Shimadzu, Tokyo, Japan). All targeted compounds were separated on a reverse phase Poroshell 120 SB C_18_ column (100 mm × 3.0 mm, 2.7 μm, Agilent Technologies, Santa Clara, CA, USA), which was eluted with a gradient mobile phase consisting of (A) water (containing 0.1% formic acid and 5 mM ammonium formate) and (B) acetonitrile. A two-step gradient elution program was utilized as follows: 0.0 min 20% B, 8.0 min 25% B, 15.0 min 60% B, 15.1 min 20% B, 18.0 min 20% B. The flow rate was set at 0.4 mL/min. The column effluent was monitored using a 4000 QTRAP^®^ LC-MS/MS (AB Sciex, Toronto, ON, Canada). 

The ESI source was operated in positive mode with the curtain, nebulizer and turbo-gas (all nitrogen) set at 20, 50 and 55 psi, respectively. The turbo-gas temperature was 500 °C and the ion spray needle voltage was 5500 V. The compound-dependent instrumental parameters of two individual precursor-to-product ion transitions specific for each analyte including precursor ion, two product ions, declustering potential (DP), entrance potential (EP), collision energy (CE) and collision cell exit potentials (CXP) were optimized and are listed in Table 3. The dwell time was 200 ms for each MRM transition.

#### 4.3.3. Method Validation

According to the Chinese Pharmacopoeia guidelines on analytical method validation [4], the method was validated in terms of linearity, sensitivity, precision (intra- and inter-day variability), repeatability, accuracy and matrix effect. Calibration curves were constructed using the peak area (Y) versus the analyte concentration (X). Each calibration curve was performed with at least six appropriate concentration levels in triplicate. Limits of detection (LODs) and quantification (LOQs) for each analyte were determined at the signal-to-noise ratio (S/N) of about 3 and 10, respectively. Precision was determined by replicated analyses (*n* = 6) of standard samples within a day (intra-day variation) and on three consecutive days (inter-day variation). The accuracy of the method was assessed by adding the targeted analytes at three different concentrations to a real sample that had previously been analyzed. The stability was evaluated by repeated analysis of the same sample solution within 48 h at room temperature. The method repeatability was determined by analyzing six independently prepared solutions of sample.

The matrix effects from endogenous substances present in the samples, especially in complex TCM matrices, may cause ion suppression or enhancement of the signal. Therefore, in this process, evaluation of the matrix effect is important and necessary. In this study, the unspiked sample, the spiked sample with addition of known amounts of standards and the standard solutions at the same concentration were analyzed as before. Each sample was prepared in triplicate, and the solution was injected into the HPLC system after filtering through a 0.22 μm microporous membrane. The matrix effect (ME, %) was calculated using the following the formula:

ME (%) = 100 × (A1 − A2)/A3

where A1 is the peak area of the analyte in the spiked sample matrix, A2 is the peak area of the analyte in the un-spiked sample matrix and A3 is the peak area of the standard at the same concentration. Generally, the ME was considered negligible when the values range from 80 to 120%.

### 4.4. Data Analysis

The statistical analysis was performed using the professional analysis software HemI version 1.0 for hierarchical clustering analysis (HCA) and OriginPro 8.5 (OriginLab Corporation, Northampton, MA, USA) for principal component analysis (PCA). HCA and PCA were used to show the unsupervised clustering pattern of the *Uncaria* species and discover the differences in samples caused by complex factors. HCA and PCA were used to discover the natural interrelationships among the chemical components for each of *Uncaria* samples.

## 5. Conclusions

In this study, an integrated evaluation system based on DNA barcoding for species identification and quantitative analysis by LC-MS/MS has been established. Twenty four batches of samples, gathered from different species and origin areas in Guangxi of China, were identified, classified and quantified successfully. The evaluation system could efficiently identify medicinal materials belong to different *Uncaria* species, and efficiently control the quality of medicinal materials from different species and origin areas. Furthermore, this system is suitable for practical use to provide a molecular identification model and quality control of *Uncaria* herbal medicines and their related products. Previous pharmacological studies demonstrated that bioactivities of the different alkaloids in these plants show significant deviations, hence, systematic identification and quality control are necessary and important to guarantee appropriate use medicinal materials of *Uncaria* for treating different illnesses, ensuring the effectiveness of the clinical application. In addition, it also provided an important reference for the establishment of the method for molecular identification and targeted quantitative analysis of medicinal materials from *Uncaria* for pharmaceutical manufacturing enterprises.

## Figures and Tables

**Table 1:** The interspecific variable sites in the ITS2 sequences of *Uncaria*.

Sample ID	Species (abbr)	Haplotypes	Variable Site/bp																
			7	23	24	25	45	59	62	63	138	145	149	158	166	196	203	219	220
UHI1	*Uncaria hirsute* (UHI)	H1	G	C	C	A	C	C	A	A	G	T	C	A	C	A	T	A	C
UHO1	*Uncaria homomalla* (UHO)	H2	*	A	*	*	T	*	*	*	*	*	*	*	*	*	*	*	*
UMA1	*Uncaria macrophylla* (UMA)	H3	*	*	*	G	T	*	T	*	*	C	*	*	*	*	*	C	*
UMA2	*Uncaria macrophylla* (UMA)	H4	*	*	*	C	T	*	T	*	*	C	*	*	*	*	*	C	*
UMA3	*Uncaria macrophylla* (UMA)	H3	*	*	*	G	T	*	T	*	*	C	*	*	*	*	*	C	*
URH1	*Uncaria rhynchophylla* (URH)	H5	*	*	T	*	T	*	*	*	*	*	T	*	*	T	*	*	*
URH2	*Uncaria rhynchophylla* (URH)	H5	*	*	T	*	T	*	*	*	*	*	T	*	*	T	*	*	*
URH3	*Uncaria rhynchophylla* (URH)	H5	*	*	T	*	T	*	*	*	*	*	T	*	*	T	*	*	*
URH4	*Uncaria rhynchophylla* (URH)	H5	*	*	T	*	T	*	*	*	*	*	T	*	*	T	*	*	*
URH5 ^a^	*Uncaria rhynchophylla* (URH)	H6	*	*	T	R	T	*	M	*	*	*	Y	*	*	W	*	M	*
URH6	*Uncaria rhynchophylla* (URH)	H5	*	*	T	*	T	*	*	*	*	*	T	*	*	T	*	*	*
URH7	*Uncaria rhynchophylla* (URH)	H5	*	*	T	*	T	*	*	*	*	*	T	*	*	T	*	*	*
URH8	*Uncaria rhynchophylla* (URH)	H5	*	*	T	*	T	*	*	*	*	*	T	*	*	T	*	*	*
URH9	*Uncaria rhynchophylla* (URH)	H5	*	*	T	*	T	*	*	*	*	*	T	*	*	T	*	*	*
URH10	*Uncaria rhynchophylla* (URH)	H5	*	*	T	*	T	*	*	*	*	*	T	*	*	T	*	*	*
URH11	*Uncaria rhynchophylla* (URH)	H5	*	*	T	*	T	*	*	*	*	*	T	*	*	T	*	*	*
URH12	*Uncaria rhynchophylla* (URH)	H5	*	*	T	*	T	*	*	*	*	*	T	*	*	T	*	*	*
URH13	*Uncaria rhynchophylla* (URH)	H5	*	*	T	*	T	*	*	*	*	*	T	*	*	T	*	*	*
URH14	*Uncaria rhynchophylla* (URH)	H5	*	*	T	*	T	*	*	*	*	*	T	*	*	T	*	*	*
URH15	*Uncaria rhynchophylla* (URH)	H5	*	*	T	*	T	*	*	*	*	*	T	*	*	T	*	*	*
USC1	*Uncaria scandens* (USC)	H7	*	*	*	*	T	T	*	*	*	*	*	*	*	*	*	*	*
USE1	*Uncaria sessilifructus* (USE)	H8	T	*	T	G	T	*	T	C	T	*	*	G	A	*	C	C	A
USI1	*Uncaria sinensis* (USI)	H9	*	*	T	G	T	*	C	*	*	*	*	*	*	*	*	C	*
USI2	*Uncaria sinensis* (USI)	H9	*	*	T	G	T	*	C	*	*	*	*	*	*	*	*	C	*

^a^ hybrid specie R = A/G, M = A/C, Y = C/T, W = A/T; * Base in this site is the same as that in the first row.

**Table 2:** Content (mg/g) of eight targeted alkaloids in materials of *Uncaria* species from Guangxi Province.

Sample ID	Species	Origin	Rhynchophylline (RC)	Isorhychophylline (IRC)	Corynoxeine (CX)	Isocorynoxeine (ICX)	Corynoxine (CN)	Corynoxine B (CNB)	Hirsutine (HTI)	Hirsuteine (HTE)	Total Contents
URH1	URH	Rong’an, Guangxi	193.8	405.0	791.9	544.1	10.0	0.0	330.8	605.1	2880.8
URH2	URH	Rong’an, Guangxi	44.0	104.2	142.5	90.4	3.0	0.0	945.7	1156.3	2485.9
URH3	URH	Rong’an, Guangxi	17.3	37.6	118.9	82.2	1.7	0.0	476.5	1090.9	1825.0
URH4	URH	Rong’an, Guangxi	12.4	27.7	64.8	43.8	1.4	0.0	422.1	912.9	1485.0
URH5	URH	Rong’an, Guangxi	18.1	40.1	51.0	33.1	1.4	0.0	146.9	145.2	435.8
URH6	URH	Rong’an, Guangxi	10.6	24.5	27.5	18.8	1.6	0.0	579.7	669.5	1332.1
URH7	URH	Xing’an, Guangxi	232.6	455.4	728.0	551.4	12.4	0.0	480.1	675.2	3135.0
URH8	URH	Lingyun, Guangxi	220.0	454.2	722.5	539.8	12.0	0.0	462.0	648.7	3059.2
URH9	URH	Yibing, Guangxi	26.6	65.0	70.1	52.7	2.9	0.0	673.9	756.6	1647.7
URH10	URH	Laibing, Guangxi	209.2	395.0	585.9	479.0	11.2	0.0	336.4	489.6	2506.3
URH11	URH	Rongshui, Guangxi	14.9	29.5	50.1	40.7	1.5	0.0	436.6	616.5	1189.9
URH12	URH	Sanjiang, Guangxi	61.6	123.4	137.4	108.3	3.3	0.0	287.5	452.7	1174.1
URH13	URH	Canwu, Guangxi	79.8	147.1	394.2	354.6	4.8	0.0	279.4	685.6	1945.4
URH14	URH	Sanjiang, Guangxi	35.7	67.0	80.2	67.8	2.8	0.0	539.9	705.5	1498.9
URH15	URH	Sanjiang, Guangxi	54.0	65.0	82.4	90.3	3.3	0.0	527.2	714.0	1536.2
UMA-1	UMA	Nanning, Guangxi	1356.0	704.1	32.4	146.2	1088.4	1212.1	32.0	14.0	4585.1
UMA-2	UMA	Nanning, Guangxi	1144.0	623.9	16.9	94.9	1057.6	1332.6	12.6	7.9	4290.4
UMA-3	UMA	Nanning, Guangxi	1612.0	1299.4	78.2	183.1	1060.4	1033.5	58.5	5.6	5330.7
USI-1	USI	Rong’an, Guangxi	7.9	4.1	0.5	0.8	5.5	4.9	1.2	1.7	26.6
USI-2	USI	Rong’an, Guangxi	22.2	11.7	0.8	1.8	16.7	17.3	1.2	1.6	73.3
USE-1	USE	Nanning, Guangxi	408.0	847.2	83.9	48.9	18.5	7.5	11.7	5.1	1430.7
UHO-1	UHO	Shanglin, Guangxi	8.0	16.7	6.5	5.0	5.5	1.1	6.0	9.2	57.9
USC-1	USC	Tianlin, Guangxi	2.9	4.4	0.6	0.4	1.5	0.8	0.8	1.0	12.4
UHI-1	UHI	Nanning, Guangxi	3.3	2.6	0.7	0.5	1.6	1.8	1.5	2.2	14.3

**Table 3:** MS parameters of the eight targeted alkaloids.

Analytes	MW.	t_R_ (min)	*m*/*z* Precursor	*m*/*z* Product ions		DP (V)	CE (eV)
RC	384.47	9.26	385	353/160.1 *^q^*		165	25/42
IRC	384.47	7.43	385	353/160.1 *^q^*		165	25/42
CX	382.45	6.65	383	267.2/160 *^q^*		160	30/40
ICX	382.45	7.83	383	267.2/160 *^q^*		160	30/40
CN	384.473	7.88	385	353/160 *^q^*		165	25/42
CNB	384.46	8.15	385	353/160 *^q^*		165	25/42
HTI	368.47	13.45	369	238/143.9 *^q^*		155	35/40
HTE	366.194	13.07	367	170.1/144 *^q^*		130	52/37
Source temperature (°C)					500		
Ionization voltage (V)					5500		
Ion source (GS1) setting (psi)					50		
Ion source (GS2) setting (psi)					55		
Curtain gas (CUR) setting (psi)					20		
Collision activation dissociation (CAD) gas setting					10		
Dwell time (ms)					200		
EP (V)					10		
CXP (V)					10		

*^q^* product ion used for quantification.

## Supplementary Materials

The following are available online at http://www.mdpi.com/1420-3049/24/1/175/s1. Figure S1: Intragenomic variation in the ITS2 sequences of Uncaria homomalla. The figure was performed by ClustalW-BOXSHADE alignment method. Black shade indicates identical base; white shade indicates variable base. Figure S2: The composition proportion of each targeted alkaloid in different Uncaria samples. Table S1: Main information of targeted alkaloids. Table S2: Method validation results of LC-MS/MS analysis.

## Abbreviations

URCU	Uncariae Ramulus Cum Uncis
URH	*Uncaria rhynchophylla*
UHI	*Uncaria hirsute*
UMA	*Uncaria macrophylla*
USI	*Uncaria sinensis*
USE	*Uncaria sessilifructus*
UHO	*Uncaria homomalla*
USC	*Uncaria scandens*
RC	rhynchophylline
IRC	isorhynchophylline
CX	corynoxeine
ICX	isocorynoxeine
CN	corynoxine
CNB	corynoxine B
HTI	hirsutine
HTE	hirsuteine
HCA	Hierarchical clustering analysis
PCA	Principal component analysis
