# Supplementary figures and images for: Molecular Identification and Targeted Quantitative Analysis of Medicinal Materials from Uncaria Species by DNA Barcoding and LC-MS/MS
Source: Molecules. 2019 Jan 4;24(1):175. doi: 10.3390/molecules24010175 (PMC6337676; doi:10.3390/molecules24010175)

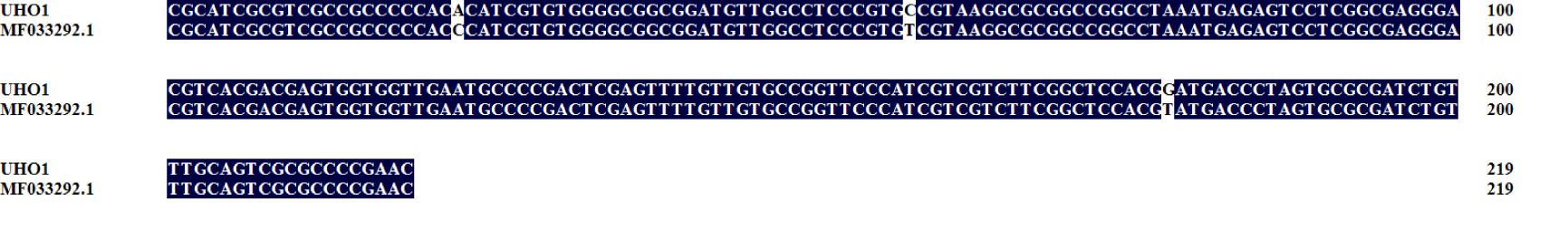

Supplement: Supplementary file 1 [file molecules-24-00175-s001.zip › molecules-405413-SI/Figure S1.tif]
